# Comparative Mitogenomics and Phylogenetic Analyses of Pentatomoidea (Hemiptera: Heteroptera)

**DOI:** 10.3390/genes12091306

**Published:** 2021-08-25

**Authors:** Shiwen Xu, Yunfei Wu, Yingqi Liu, Ping Zhao, Zhuo Chen, Fan Song, Hu Li, Wanzhi Cai

**Affiliations:** 1 Department of Entomology and MOA Key Lab of Pest Monitoring and Green Management, College of Plant Protection, China Agricultural University, Beijing 100193, China; xusw2019@126.com (S.X.); wuyunfeiakx@126.com (Y.W.); yingqiliu0720@163.com (Y.L.); insectchen625@126.com (Z.C.); fansong@cau.edu.cn (F.S.); tigerleecau@hotmail.com (H.L.); 2 Key Laboratory of Environment Change and Resources Use in Beibu Gulf (Ministry of Education) and Guangxi Key Laboratory of Earth Surface Processes and Intelligent Simulation, Nanning Normal University, Nanning 530001, China; zpyayjl@126.com

**Keywords:** Pentatomoidea, mitochondrial genome, sequence heterogeneity, phylogeny

## Abstract

Pentatomoidea is the largest superfamily of Pentatomomorpha; however, the phylogenetic relationships among pentatomoid families have been debated for a long time. In the present study, we gathered the mitogenomes of 55 species from eight common families (Acanthosomatidae, Cydnidae, Dinidoridae, Scutelleridae, Tessaratomidae, Plataspidae, Urostylididae and Pentatomidae), including 20 newly sequenced mitogenomes, and conducted comparative mitogenomic studies with an emphasis on the structures of non-coding regions. Heterogeneity in the base composition, and contrasting evolutionary rates were encountered among the mitogenomes in Pentatomoidea, especially in Urostylididae, which may lead to unstable phylogenetic topologies. When the family Urostylididae is excluded in taxa sampling or the third codon positions of protein coding genes are removed, phylogenetic analyses under site-homogenous models could provide more stable tree topologies. However, the relationships between families remained the same in all PhyloBayes analyses under the site-heterogeneous mixture model CAT + GTR with different datasets and were recovered as (Cydnidae + (((Tessaratomidae + Dinidoridae) + (Plataspidae + Scutelleridae)) + ((Acanthosomatidae + Urostylididae) + Pentatomidae)))). Our study showed that data optimizing strategies after heterogeneity assessments based on denser sampling and the use of site-heterogeneous mixture models are essential for further analysis of the phylogenetic relationships of Pentatomoidea.

## 1. Introduction

Pentatomoidea (Hemiptera: Pentatomomorpha), also known as stink bugs and their relatives, is the largest superfamily within Pentatomomorpha, with over 8000 species [1,2]. Most taxa in this widespread terrestrial superfamily are phytophagous, feeding on a variety of fruits, vegetables, grain crops or wild plants, and thus some are economically important pests in agriculture and forestry [2,3].

Although the monophyly of Pentatomoidea has been supported by both morphological and molecular data [4,5,6,7,8,9,10], relationships at the family level remain controversial. For instance, there has been a longstanding debate on how many valid families are present in Pentatomoidea (see summaries by Grazia et al. [4] and Rider [2]) The division of Pentatomoidea into 18 families was only generally accepted recently, with a few doubts yet to be addressed [1]. Using morphological characters, Gapud [5] and Xu [11] were the first ones to study the phylogeny of Pentatomoidea. Following their studies, phylogenetic analyses based on molecular data, including nuclear and mitochondrial genes fragments [8,12,13,14], or whole mitochondrial genomes (mitogenomes) [7,9,10] were subsequently performed. More recently, a phylogenetic study on Pentatomoidea combining morphological and molecular data was performed by Grazia et al. [4]. The above-mentioned studies, despite having different resulting topologies, reached agreement on relationships among certain lineages. For instance, the sister relationship between Tessaratomidae and Dinidoridae was supported by the majority of these phylogenetic studies. The sister relationship between Urostylididae and Acanthosomatidae was also recovered by different molecular and morphological markers [8,9,11,15]. In addition, mitogenomic analyses performed by different research groups have independently demonstrated that Urostylididae, or the clade involving Urostylididae is the earliest branching lineage of Pentatomoidea [7,10,13,14].

The mitogenome is one of the most widely used molecular markers in insect phylogenetic studies, but the compositional heterogeneity and accelerated evolutionary rates of the mitogenomes in certain groups may result in erroneous grouping of unrelated taxa [16,17,18,19]. Recent studies have demonstrated that the site-heterogeneous mixture model implemented in the software PhyloBayes may help in reducing the susceptibility of analyses to heterogeneity and thus provide more stable topologies [16,17,19,20,21]. Moreover, denser taxa sampling could be another strategy to assess the potential impact of substitutional and compositional biases, and to reduce tree reconstruction artifacts [9,22,23]. However, in regard to Pentatomoidea, some previous phylogenetic analyses had limited taxon sampling with only one species per family, hindering comprehensive data analysis [4,7,12].

So far, phylogenetic studies of Pentatomoidea using mitogenomic data are mainly based on sequences of eight common families (Acanthosomatidae, Cydnidae, Dinidoridae, Scutelleridae, Tessaratomidae, Plataspidae, Urostylididae and Pentatomidae) deposited in GenBank. In the present study, we gathered mitogenomic sequences of 55 species from these eight families, including 20 newly generated mitogenomes to better analyze the impact of sequence heterogeneity. In order to assess better strategies to resolve the phylogeny of this superfamily, we compared the effects of different models for phylogenetic analysis (i.e., site-homogeneous and site-heterogeneous mixture model), as well as some data optimizing methods, on the quality of resulting topology. Based on our analysis, we discuss potential ways to enhance future phylogenetic analysis on this highly diverse insect group.

## 2. Materials and Methods

### 2.1. Sample Collection and DNA Extraction

Voucher specimens of 20 pentatomoid species were collected in the field, preserved in anhydrous ethanol, and stored at −20 °C at the Entomological Museum of China Agricultural University. Detailed collection information is provided in Table S1. Genomic DNA of specimens was extracted from thorax muscle tissues using the DNeasy Blood and Tissue Kit (Qiagen, Venlo, The Netherlands) as per the manufacturer’s protocol.

### 2.2. Mitochondrial Genome Sequencing and Assembly

In order to improve sequencing efficiency and avoid resource waste, hybrid libraries were adopted according to Gillett et al. [22]. All Illumina TruSeq libraries were prepared with an average insert size of 350 bp and sequenced using the Illumina HiSeq 2500 platform (Illumina, San Diego, CA, USA) with 150 bp paired-end reads. Prinseq version 0.20.4 (Edwards Lab, San Diego, CA, USA) [24] was used to remove short and low-quality reads with poly-Ns (>15 bp Ns), or >75 bp bases with a quality score ≤ 3. The remaining reads were de novo assembled using IDBA-UD [25], with minimum and maximum k values of 45 and 145 bp, respectively. The partial sequences of *COI* and *srRNA* of each species were obtained by standard PCR reaction and Sanger sequencing to identify the corresponding mitogenome assemblies. Clean reads were mapped using Geneious version 10.1.3 (http://www.geneious.com/ (accessed on 22 December 2020)) [26] to evaluate assembly accuracy.

### 2.3. Mitochondrial Genome Annotation and Sequence Analysis

Gene sequences were annotated using MitoZ [27], and further corrected in Geneious. The locations and secondary structures of tRNA genes were determined using tRNAscan-SE version 1.21 [28] and ARWEN version 1.2 [29]. Protein-coding genes (PCGs) and rRNA genes were identified by aligning their sequences with those of homologous genes of other Pentatomoidea species. Nucleotide composition of mitogenomes and the codon usage of PCGs were analyzed with MEGA version 7.0. (IGEM, Philadelphia, PA, USA) [30] Nucleotide compositional differences were calculated using the following formulae: AT skew = (A − T)/(A + T) and GC skew = (G − C)/(G + C) [31]. DnaSP 5.0 [32] was used to calculate the rate of synonymous substitutions (Ks) and the rate of non-synonymous substitutions (Ka) for PCGs.

### 2.4. Phylogenetic Analysis

The 13 PCGs of each species were separately aligned using the L-INS-I strategy in the MAFFT algorithm [33], which was implemented in TranslatorX [34]. Two rRNA genes were aligned individually using the G-INS-I strategy in MAFFT version 7.0 (iFReC, Osaka, Japan) [35]. All alignments were manually checked using MEGA. Gene fragments were imported into Geneious and concatenated into two datasets: (1) the PCGRNA matrix with 12,311 nucleotides, corresponding to 13 PCGs and two rRNA genes; and (2) the PCG12RNA matrix with 8840 nucleotides, corresponding to the first and second codon positions of the 13 PCGs and two rRNA genes. Heterogeneity of sequence divergence within the datasets (i.e., PCGRNA and PCG12RNA) was analyzed using AliGROOVE [36] with the default sliding window size. This metric established pairwise sequence distances between individual terminals or subclades with terminals outside of the focal group. The distances were then compared to distances over the entire data matrix. The metric values can vary between −1, if distances are very different from the average for the entire data matrix, to +1, for distances which match the average for the entire matrix.

PartitionFinder2 [37] was used to select the optimal partitioning schemes and substitution models for analyses under site-homogeneous models. We used the “greedy” algorithm with branch lengths estimated as “unlinked” and the Akaike information criterion. The input configuration file containing 15 initial gene partitions (13 PCGs and two rRNA genes) was created and the optimal partitioning schemes for each dataset are shown in Table S2. The site-homogeneous model GTR + I + G was determined to be the most suitable model for all partitions in maximum likelihood (ML) analyses using IQ-TREE web server [38] and Bayesian analyses using MrBayes version 3.2.7 [39]. For IQ-TREE, phylogenetic trees were conducted with 1000 SH-aLRT replicates. For MrBayes, two simultaneous runs of 10 million generations were conducted for the datasets, and trees were sampled every 1000 generations, with the first 25% discarded as burn-in. Stationarity was considered to be reached when the average standard deviation of split frequencies was below 0.01. Bayesian trees were also constructed using PhyloBayes MPI version 1.5a (Robert Cedergren Centre, Montreal, PQ, Canada) [40] under the site-heterogeneous mixture model CAT + GTR [41,42]. The Markov chain Monte Carlo chains were run independently after removing constant sites from the alignment, and they were stopped after the two runs had satisfactorily converged (i.e., maxdiff < 0.3). A consensus tree was produced from the remaining trees after discarding the initial 25% of trees of each run as burn-in.

Phylogenetic analysis was first performed using whole mitogenomes of the 55 species (Table 1). Two species of Pyrrhocoroidea and two species of Aradoidea (Table 1) were selected as outgroups. Because the mitogenomes of Urostylididae showed the highest heterogeneity (see Seciton 3 below) among eight families, we performed another phylogenetic analysis following the same setting but excluding this family to assess the effect of heterogeneity on the resulting topology.

## 3. Results and Discussion

### 3.1. General Features of Pentatomoid Mitogenomes

Among the 55 species involved in the comparative analyses, 44 have complete mitogenomes. A total of 37 typical genes (i.e., 13 PCGs, 2 rRNAs, and 22 tRNAs) and a control region were present in all 55 of the pentatomoid species, as has been reported in most insects [43,44], and no gene rearrangement occurred. There were three conservative gene overlaps within Pentatomoidea, namely an 8 bp overlap between *trnW* and *trnC*, a 7 bp overlap between *ATP8* and *ATP6*, and a 7 bp overlap between *ND4* and *ND4L*. The largest intergenic spacer was located between *trnS2* and *ND1*. Notably, similar overlaps and spacer regions are commonly in other insects of the suborder Heteroptera [43].

**Figure 1:**
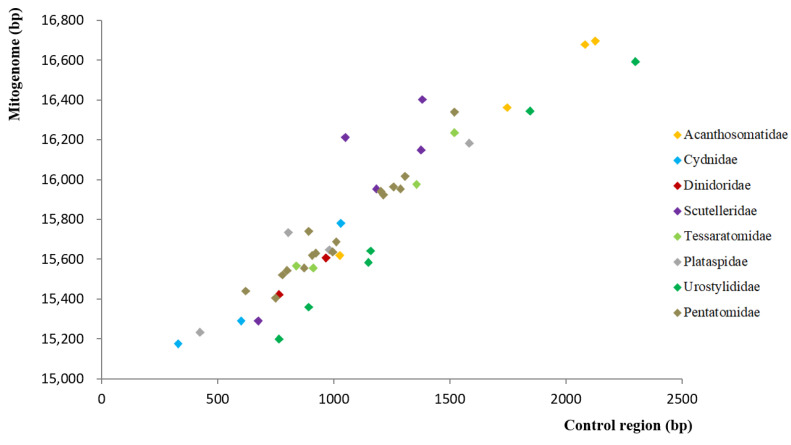
Size of the complete mitogenomes and complete control regions of 44 Pentatomoidea species.

The length of the 44 complete mitogenomes ranged from 15,173 bp (i.e., Cydnidae sp.) to 16,694 bp (*Anaxandra taurina*), with an average size of 15.8 kb. The observed length variation among mitogenomes can be mostly attributed to the length variation in control regions (Figure 1). Furthermore, all 55 mitogenomes displayed a strong AT nucleotide bias with the A + T content ranging from 67.2% (*Scoparipes salvazai*) to 78.4% (*Gonopsis affinis*). A positive AT skew (i.e., 0.069 to 0.251) and a negative GC skew (i.e., −0.255 to −0.100) were observed in all 55 Pentatomoidea species, which are common in insect mitogenomes [45,46,47].

Most PCGs began with a typical ATN codon (i.e., ATA/ATT/ATG/ATC), while the start codon of *COI* was TTG in all pentatomoid species, and a small number of start codons were GTG in *ATP8* and *ATP6*. As for stop codons, they mostly consisted of TAA or TAG, while a single T residue was used as an incomplete stop codon in *COI*, *COII*, *ATP6*, *COIII*, *ND3*, *ND5*, *ND6*, and *ND1*, which has also been detected in other hemipterans [48,49]. In particular, the stop codons of *COII* were all incomplete, except for that in *Mattiphus splendidus*.

The length of tRNA genes ranged from 59 to 76 bp. With the exception of *trnS1* and *trnV*, most tRNA genes could be folded into the typical clover leaf secondary structure. The dihydrouridine (DHU) arm of *trnS1* was a simple loop in 47 of the 55 examined pentatomoid species, whereas the DHU arm of *trnV* formed the same loop structure in 18 species. Furthermore, the sizes of *lrRNA* and *srRNA* genes ranged from 1238 (*Urolabida histrionica*) to 1387 bp (*S. salvazai*) and from 753 (*A. taurina*) to 878 bp (*Calacta lugubris*), respectively. The average A + T content of *lrRNA* genes was 77.9%, and that of *srRNA* genes was 76.4%.

### 3.2. Non-Coding Regions (NCR) of Pentatomoid Mitogenomes

**Figure 2:**
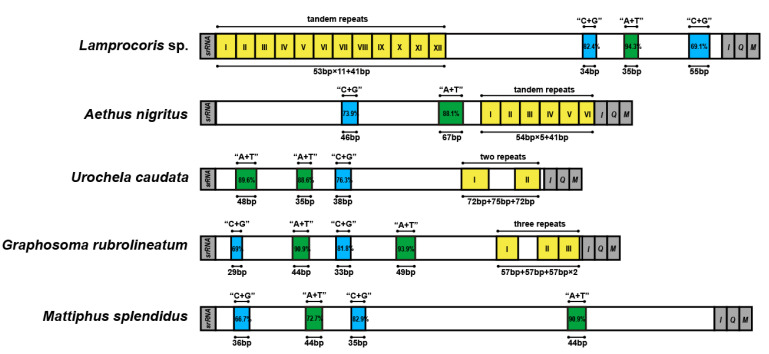
Control regions of the representative pentatomoid mitogenomes. Structure elements found in the control region are labeled with different color blocks; green: A + T rich sequence block; blue: C + G rich sequence block; yellow with roman numerals inside: repeat sequences; grey: control regions flanking genes *srRNA*, *trnI*, *trnQ*, or *trnM*.

The control regions (CR) were the NCRs with the greatest length variation, and total lengths of CR ranged from 334 (*S. salvazai*) to 2302 bp (*Urochela quadrinotata*) among the 44 complete pentatomoid mitogenomes. The general structures of the CR of these 44 species are shown in Figure S1. Three kinds of structural elements were summarized: A + T rich sequence blocks, C + G-rich sequence blocks, and repeat sequences. The arrangement and sequences of the A + T-rich and C + G-rich blocks were relatively conserved within the family Pentatomidae—that is, a C + G-rich region of approximately 30 bp and an A + T rich region of approximately 45 bp appeared alternately. Except for four species (i.e., *Eusthenes cupreus*, *M. splendidus* (Figure 2), *S. salvazai*, and *Zicrona caerulea*) that had no repeat sequences in their CR, tandem and non-tandem repeats were abundant among the pentatomoid species that were assessed. In particular, CRs of 30 species contained only one type of repeat unit, while those of the remaining 10 species contained two or three types.

CRs that contained only one type of tandem repeat unit were present in 16 species, and the repeat region was located at either the start (e.g., *Lamprocoris* sp.) or the end (e.g., *Aethus nigritus*) of CR (Figure 2). The tandem repeat units were no more than 150 bp in length, and most of them consisted of small fragments of approximately 50 bp in length. Notably, CRs with more than 10 tandem units occurred in Acanthosomatidae, Urostylididae, Scutelleridae, and Pentatomidae. Apart from Cydnidae, the other seven families showed CRs that contained non-tandem repeats, which could be divided into two types: completely disconnected and with only two units (e.g., *Urochela caudata*; Figure 2), or partially disconnected tandem repeats with at least two units connected to each other (e.g., *Graphosoma rubrolineatum*; Figure 2). Partially disconnected tandem repeats were located in the CR of Acanthosomatidae, Urostylididae, Scutelleridae, Pentatomidae, and Plataspidae. The intervals between repeats were generally less than 200 bp, although considerably long intervals were observed in *C. lugubris* (531 bp) and *Scotinophara lurida* (467 bp).

In addition to the CR, another long NCR was located between *trnS2* and *ND1* in 50 of the 55 species of Pentatomoidea, and it was 30 bp long on average. Notably, there was a 16 bp region with 84.2% similarity in this NCR.

### 3.3. Sequence Heterogeneity in Pentatomoid Mitogenomes and Phylogenetic Analyses

**Figure 3:**
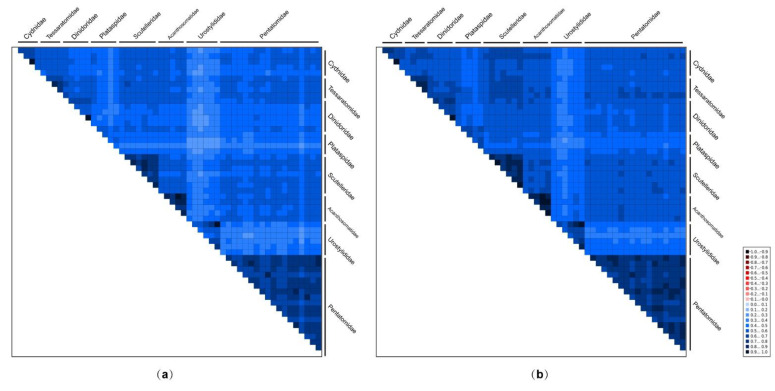
AliGROOVE analysis of 55 pentatomoid species based on PCGRNA (**a**) and PCG12RNA (**b**). The mean similarity score between sequences is represented by colored squares, based on AliGROOVE scores ranging from −1, which indicates a great difference in rates from the remainder of the data set, that is, heterogeneity (red coloring), to +1, which indicates rates that matched all other comparisons (blue coloring).

The AliGROOVE analyses indicated that the degree of heterogeneity of the PCG12RNA dataset (mean similarity score, 0.274) was lower than that of the PCGRNA dataset (0.236) (Figure 3). The third codon positions showed higher heterogeneity (lowest similarity score, 0.013) than the first codon positions (0.255) and the second codon positions (0.360) (Figure S2). In addition, the heterogeneity in sequence divergence was stronger for Urostylididae (mean similarity scores: 0.181 for PCGRNA dataset and 0.220 for PCG12RNA dataset) than for other pentatomoid families (0.327 for PCGRNA dataset and 0.376 for PCG12RNA dataset) (Figure 3). Previous studies have demonstrated that the compositional heterogeneity of mitogenomes in certain groups may result in the erroneous grouping of unrelated taxa [16,17,18,19], so Urostylididae may be unstably placed or misplaced in phylogenetic trees as a result of the divergences between these taxa.

**Figure 4:**
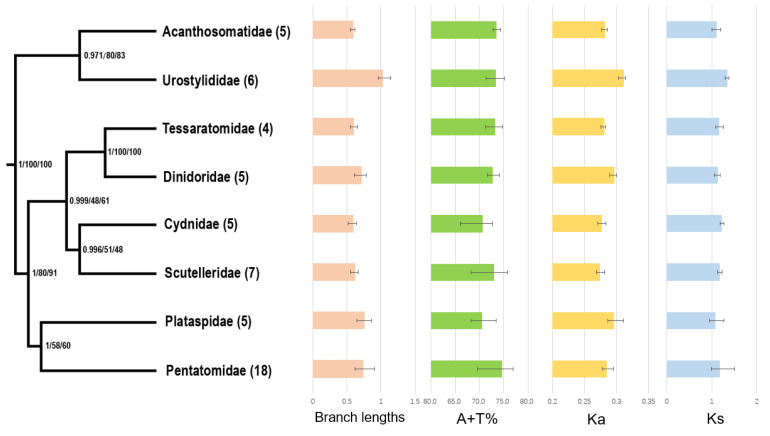
Systematic errors in phylogenetic analyses with site-homogeneous models. Supports at nodes (from left to right) obtained via MrBayes based on PCGRNA, IQ-TREE based on PCGRNA and IQ-TREE based on PCG12RNA. Numbers in brackets indicate the number of species in the corresponding family that were used for phylogenetic analysis. The branch lengths were calculated by MrBayes based on PCGRNA datasets. The A + T content (%), rate of non-synonymous substitutions (*Ka*), and rates of synonymous substitutions (*Ks*) were calculated from the protein-coding genes. Error bars represent standard deviations from the data of multiple species.

Our phylogenetic analyses consistently supported the monophyly of Pentatomoidea and each of the sampled families. The results of the MrBayes analysis with the PCGRNA dataset (Figure S3) and the ML analyses with the PCGRNA and PCG12RNA datasets (Figure S4) under the site-homogeneous models show the same topology (Figure 4). The eight major families of Pentatomoidea formed four sister groups: (Acanthosomatidae + Urostylididae), (Tessaratomidae + Dinidoridae), (Cydnidae + Scutelleridae) and (Plataspidae + Pentatomidae), but support values in ML analyses of the latter two were relatively low (i.e., <70). Comparable to many previous phylogenetic studies [7,10,13,14], the clade involving Urostylididae was found to be the earliest branching lineage. The sister groups of (Cydnidae + Scutelleridae) and (Plataspidae + Pentatomidae) were also recovered by Zhao et al. [10] and Liu et al. [9] using mitochondrial sequences, respectively, but with relatively limited sampling and low support.

We calculated the A + T content in the whole mitogenomes as well as *Ka* and *Ks* of the 13 PCGs of each taxon, and then mapped the values onto the consensus tree (Figure 4). The A + T content in Pentatomidae (74.7% ± 3.7%) was higher than that in other families, and the lowest A + T content values were present in Plataspidae (70.5% ± 2.6%) and Cydnidae (70.6% ± 3.3%). Furthermore, an accelerated evolutionary rate was observed in Urostylididae. The average *Ka* (0.311) and *Ks* (1.351) in Urostylididae were significantly higher than those of other families, and its branch length (1.021) was the longest. The compositional biases and accelerated evolutionary rates of pentatomoid mitogenomes may be the reason for the unstable phylogenetic relationships and low nodal support values among the corresponding families. After removing Urostylididae from taxa sampling, all MrBayes and ML trees showed a different topology among families (Figures S5 and S6): Acanthosomatidae remained the earliest branching lineage, but the remaining taxa were recovered as (Pentatomidae + (Cydnidae + ((Tessaratomidae + Dinidoridae) + (Plataspidae + Scutelleridae)))). Coincidently, when removing the third codon positions of PCGs, the MrBayes analysis involving eight families based on PCG12RNA dataset showed the same relationships as above (Figure S7). These results show that sequences with high heterogeneity could affect the stability of tree topologies when using the site-homogeneous model.

**Figure 5:**
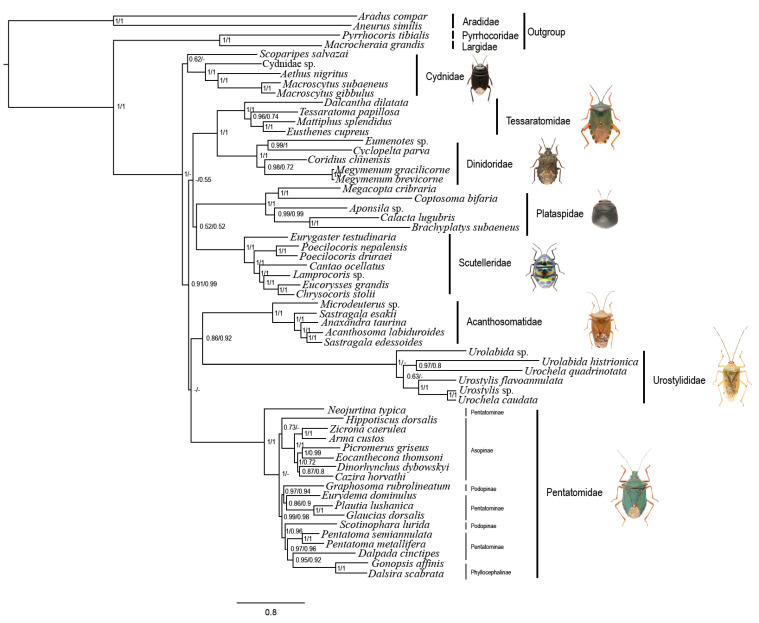
Phylogenetic tree of Pentatomoidea inferred via Bayesian analyses based on the PCGRNA and PCG12RNA datasets. The nodal support indicates the Bayesian posterior probabilities of PCGRNA/PCG12RNA. “-” indicates node support values below 0.50.

The topology constructed by PhyloBayes analyses under the site-heterogeneous mixture model CAT + GTR (Figure 5) differed from that of the ML and MrBayes analyses. Cydnidae were the earliest branching lineage of the tree, and the other seven families were divided into two lineages: one was ((Tessaratomidae + Dinidoridae) + (Plataspidae + Scutelleridae)), while the other was ((Acanthosomatidae + Urostylididae) + Pentatomidae)). The remaining topology remained the same when Urostylididae was excluded, whereas the support values of the relationships among most of the families increased (Figure S8). Notably, the clade ((Tessaratomidae + Dinidoridae) + (Plataspidae + Scutelleridae)) was also supported by the analyses under site-homogeneous models without Urostylididae in sampling. Consistent with previous phylogenetic analyses on Coleoptera [17], Holometabola [18] and Heteroptera [19], our results show that the site-heterogeneous mixture model can reduce system errors and provide more stable phylogenetic relationships. However, the relatively low support values of some family-level relationships indicated that using mitochondrial data only may not be sufficient to reconstruct the phylogenetic relationships between pentatomoid families, and combining other kinds of data such as nuclear genes and morphological characters is probably needed in future analyses.

## 4. Conclusions

Based on a dense taxa sampling, we discovered strong heterogeneity in terms of base composition as well as contrasting evolutionary rates among the pentatomoid mitogenomes. Our study demonstrated that denser sampling, data optimizing (e.g., removing taxa with high heterogeneity and/or the third codon positions of PCGs) and the use of the appropriate evolutionary model (e.g., the site-heterogeneous mixture model CAT + GTR) are necessary strategies for better resolving the family-level phylogeny of Pentatomoidea in future studies.

## Figures and Tables

**Table 1:** Taxa used in the present study.

Superfamily	Family	Species	GenBank Accession Number
Aradoidea	Aradidae	*Aneurus similis*	NC_030360
		*Aradus compar*	NC_030362
Pyrrhocoroidea	Largidae	*Macrocheraia grandis*	MK809515
	Pyrrhocoridae	*Pyrrhocoris tibialis*	KX355214
Pentatomoidea	Acanthosomatidae	*Microdeuterus* sp. ^a^	MW847242
		*Sastragala esakii* ^a^	MW847247
		*Acanthosoma labiduroides*	JQ743670
		*Anaxandra taurina*	NC_042801
		*Sastragala edessoides*	JQ743676
	Cydnidae	*Aethus nigritus* ^a^	MW847231
		*Macroscytus subaeneus* ^a^	MW847241
		Cydnidae sp.	MH643815
		*Macroscytus gibbulus*	NC_012457
		*Scoparipes salvazai*	NC_042800
	Dinidoridae	*Eumenotes* sp. ^a^	MW847237
		*Megymenum gracilicorne*	NC_042810
		*Cyclopelta parva*	NC_037739
		*Coridius chinensis*	JQ739179
		*Megymenum brevicorne*	JQ739181
	Scutelleridae	*Chrysocoris stolii* ^a^	MW847234
		*Poecilocoris druraei* ^a^	MW847246
		*Cantao ocellatus*	NC_042803
		*Eurygaster testudinaria*	NC_042808
		*Eucorysses grandis*	JQ743671
		*Lamprocoris* sp.	JQ743674
		*Poecilocoris nepalensis*	JQ743675
	Tessaratomidae	*Dalcantha dilatata* ^a^	MW847235
		*Mattiphus splendidus*	MN496304
		*Eusthenes cupreus*	NC_022449
		*Tessaratoma papillosa*	NC_037742
	Plataspidae	*Brachyplatys subaeneus* ^a^	MW847232
		*Calacta lugubris* ^a^	MW847233
		*Aponsila* sp.	MF497710
		*Coptosoma bifaria*	NC_012449
		*Megacopta cribraria*	NC_015342
	Urostylididae	*Urochela caudata* ^a^	MW847248
		*Urolabida histrionica* ^a^	MW847249
		*Urochela quadrinotata*	NC_020144
		*Urolabida* sp.	MF497734
		*Urostylis flavoannulata*	NC_037747
		*Urostylis* sp.	JQ743679
	Pentatomidae	*Dalpada cinctipes* ^a^	MW847236
		*Eurydema dominulus* ^a^	MW847238
		*Glaucias dorsalis* ^a^	MW847239
		*Hippotiscus dorsalis* ^a^	MW847240
		*Neojurtina typica* ^a^	MW847243
		*Pentatoma metallifera* ^a^	MW847244
		*Plautia lushanica* ^a^	MW847245
		*Zicrona caerulea* ^a^	MW847250
		*Arma custos*	MT535604
		*Cazira horvathi*	NC_042817
		*Dalsira scabrata*	NC_037374
		*Dinorhynchus dybowskyi*	NC_037724
		*Eocanthecona thomsoni*	NC_042816
		*Gonopsis affinis*	NC_036745
		*Graphosoma rubrolineatum*	NC_033875
		*Pentatoma semiannulata*	MT985377
		*Picromerus griseus*	NC_036418
		*Scotinophara lurida*	NC_042815

^a^ Mitogenomes sequenced in the present study.

## Data Availability

All mitochondrial genome sequences used in this study have been deposited into GenBank (accession numbers: MW847231–MW847250).

## Supplementary Materials

The following are available online at https://www.mdpi.com/article/10.3390/genes12091306/s1, Figure S1: Control regions of the 44 complete pentatomoid mitogenomes; Figure S2: AliGROOVE analysis of 55 pentatomoid species based on first codon positions of PCGs (a), second codon positions of PCGs (b) and third codon positions of PCGs (c); Figure S3: Phylogenetic tree of Pentatomoidea inferred via MrBayes based on the PCGRNA dataset; Figure S4: Phylogenetic tree of Pentatomoidea inferred via IQ-TREE based on the PCGRNA and PCG12RNA datasets; Figure S5: Phylogenetic tree of Pentatomoidea (excluding Urostylididae) inferred via MrBayes based on the PCGRNA and PCG12RNA datasets; Figure S6: Phylogenetic tree of Pentatomoidea (excluding Urostylididae) inferred via IQ-TREE based on the PCGRNA and PCG12RNA datasets; Figure S7: Phylogenetic tree of Pentatomoidea inferred via MrBayes based on the PCG12RNA dataset; Figure S8: Phylogenetic tree of Pentatomoidea (excluding Urostylididae) inferred via PhyloBayes based on the PCGRNA and PCG12RNA datasets; Table S1: Collection information of taxa used in the present study; Table S2: Partition strategies used in phylogenetic analyses under site-homogeneous model.
