# Supplementary material for: Comparative Mitogenomics and Phylogenetic Analyses of Pentatomoidea (Hemiptera: Heteroptera)
Source: Genes (Basel). 2021 Aug 25;12(9):1306. doi: 10.3390/genes12091306 (PMC8471585; doi:10.3390/genes12091306)
Supplement: Supplementary file 1 [file genes-12-01306-s001.zip › genes-1325796-supplementary.pdf]

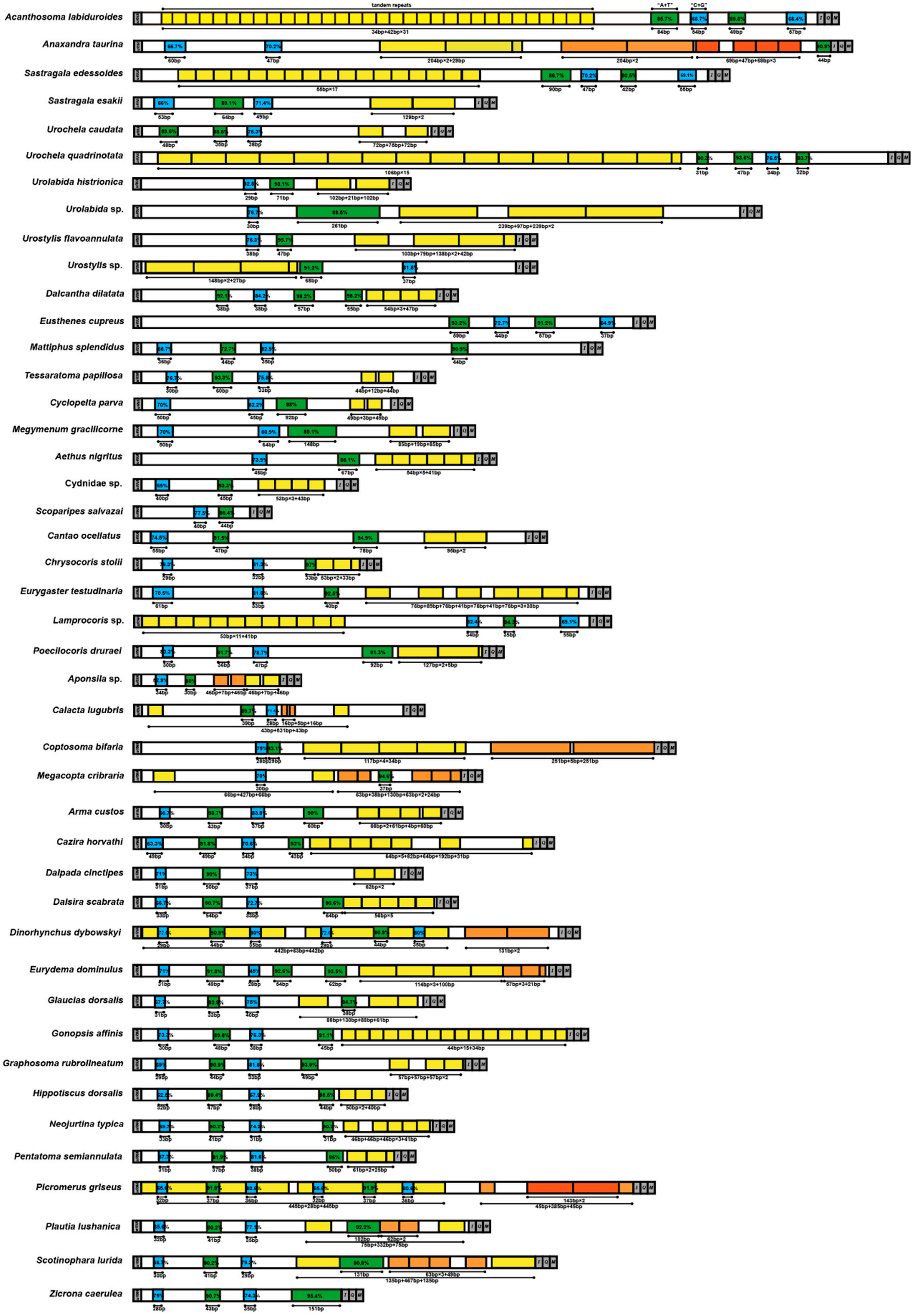

Figure S1. Control regions of the 44 complete pentatomoid mitogenomes. Structure elements present in the control

region are labeled with different color blocks; green: A + T rich sequence block; blue: C + G rich sequence block; yellow, orange, or red: repeat sequences; grey: control regions that flank genes *srRNA*, *trnI*, *trnQ*, and *trnM*.

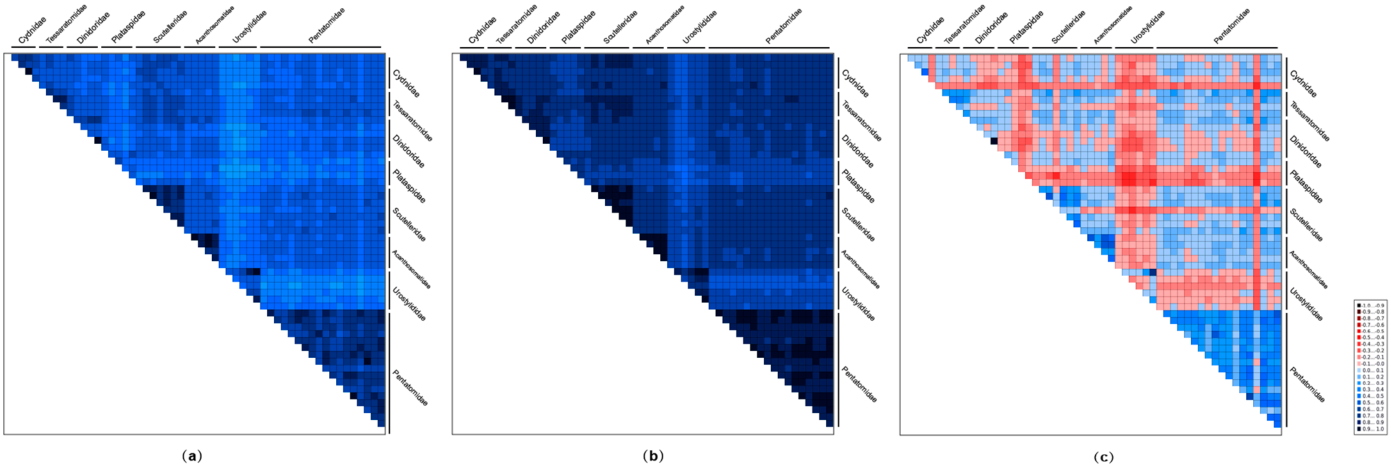

Figure S2. AliGROOVE analysis of 55 pentatomoid species based on first codon positions of PCGs (a), second codon positions of PCGs (b) and third codon positions of PCGs (c). The mean similarity score between sequences is represented by colored squares, based on AliGROOVE scores ranging from -1, which indicates a great difference in rates from the remainder of the data set, that is, heterogeneity (red coloring), to +1, which indicates rates that matched all other comparisons (blue coloring).

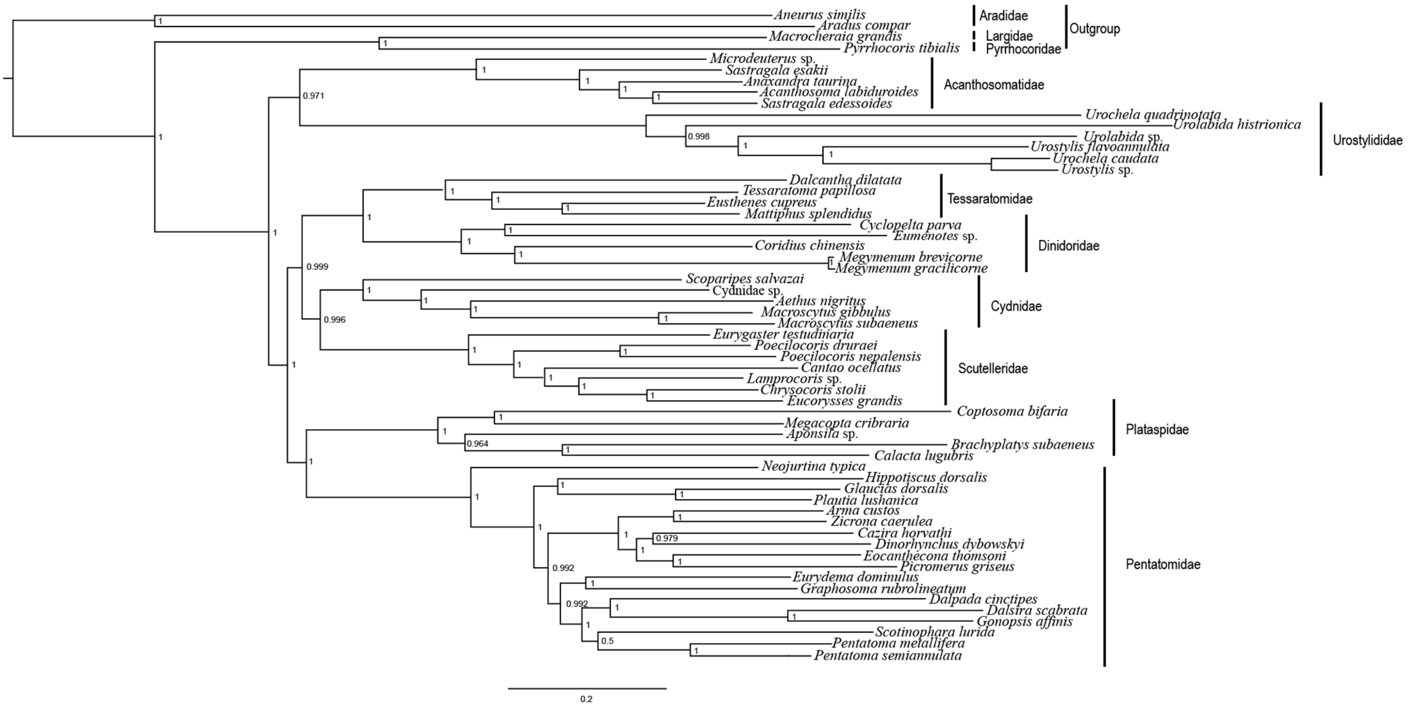

Figure S3. Phylogenetic tree of Pentatomoidea inferred via MrBayes based on the PCGRNA dataset. The nodal support indicates the Bayesian posterior probabilities.

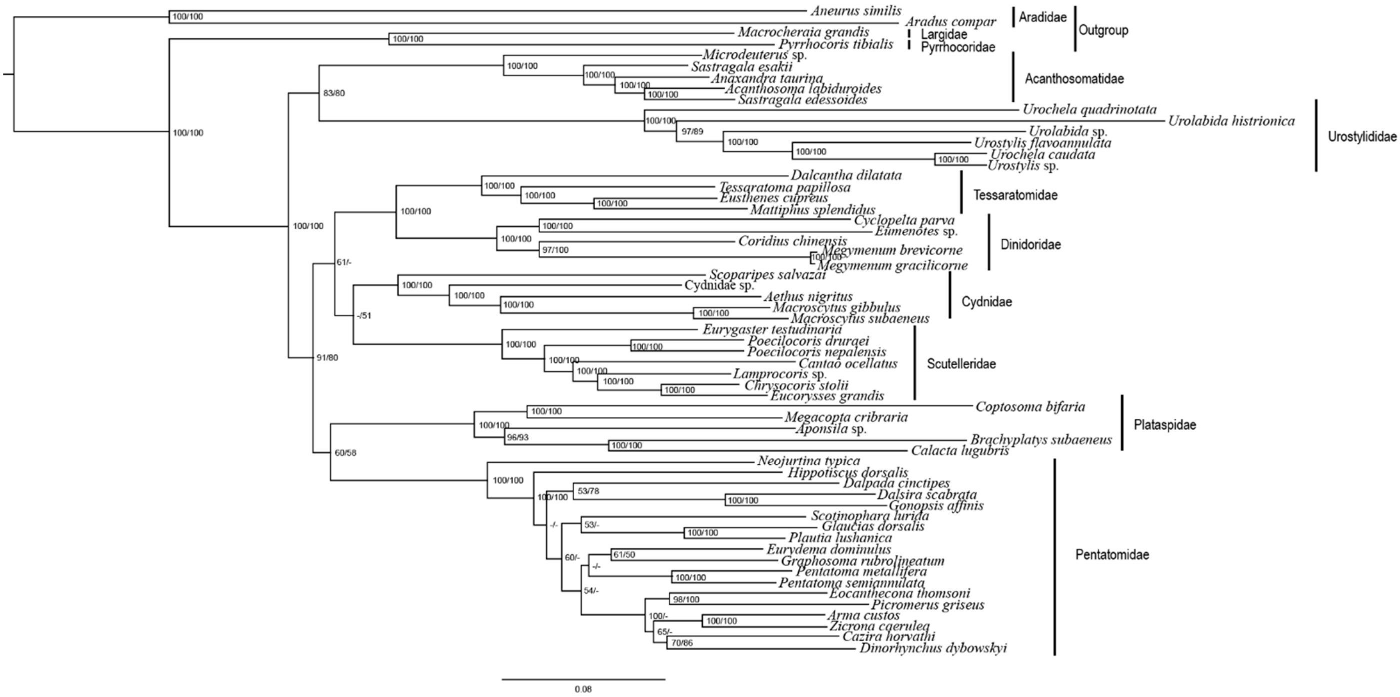

Figure S4. Phylogenetic tree of Pentatomoidea inferred via IQ-TREE based on the PCGRNA and PCG12RNA datasets. The nodal support indicates the bootstrap support values of PCG12RNA/PCGRNA. “-” indicates node support values below 50.

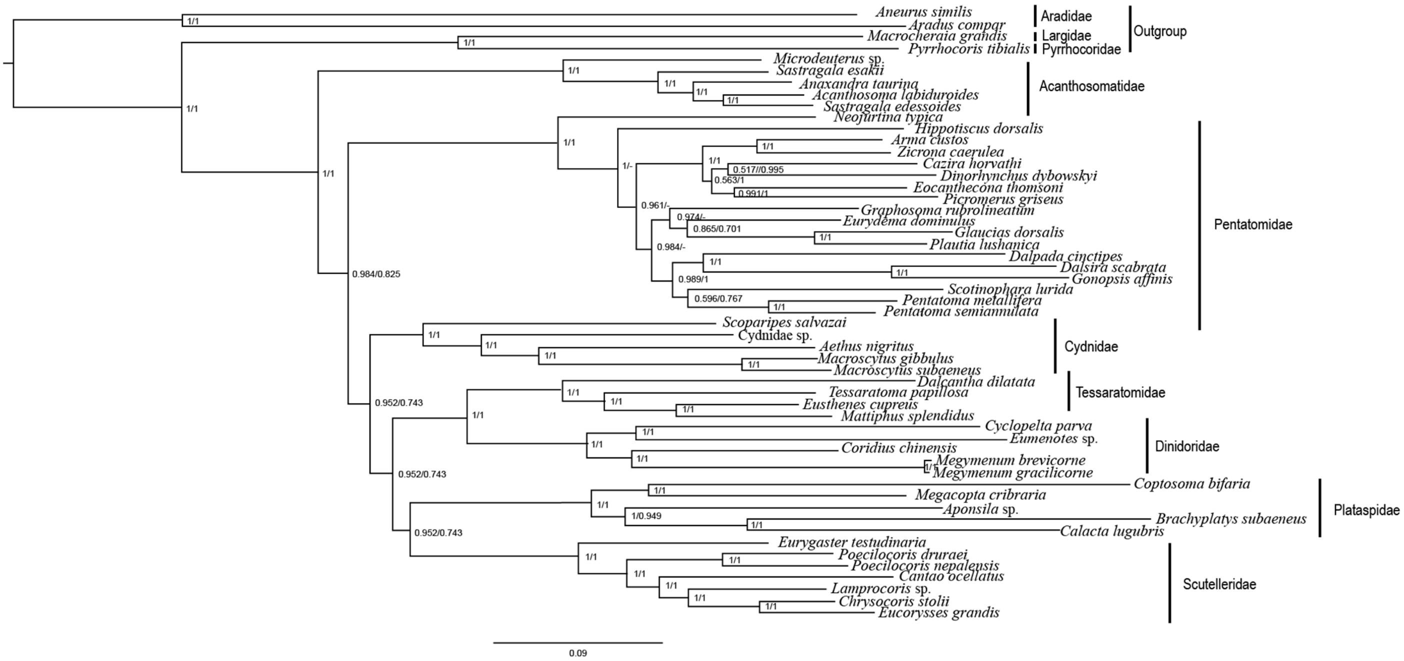

Figure S5. Phylogenetic tree of Pentatomoidea (excluding Urostylididae) inferred via MrBayes based on the PCGRNA and PCG12RNA datasets. The nodal support indicates the Bayesian posterior probabilities of PCG12RNA/PCGRNA. “-” indicates node support values below 0.50.

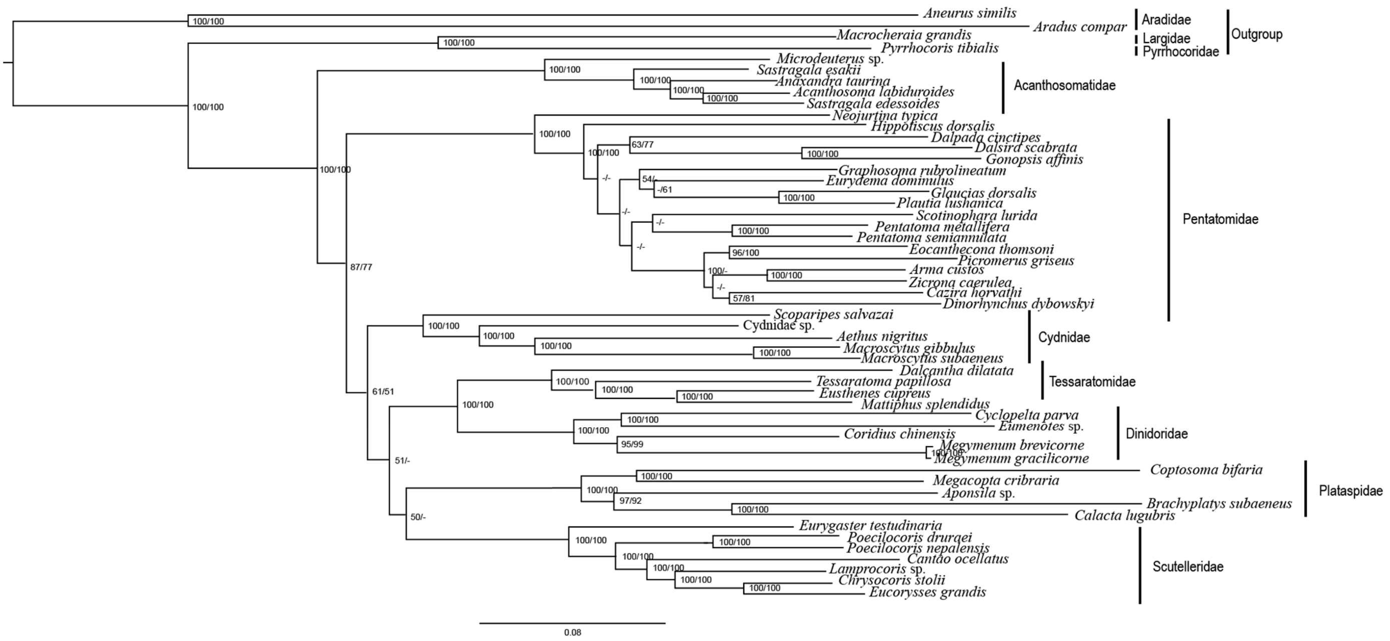

Figure S6. Phylogenetic tree of Pentatomoidea (excluding Urostylididae) inferred via IQ-TREE based on the PCGRNA and PCG12RNA datasets. The nodal support indicates the bootstrap support values of PCG12RNA/PCGRNA. “-” indicates node support values below 50.

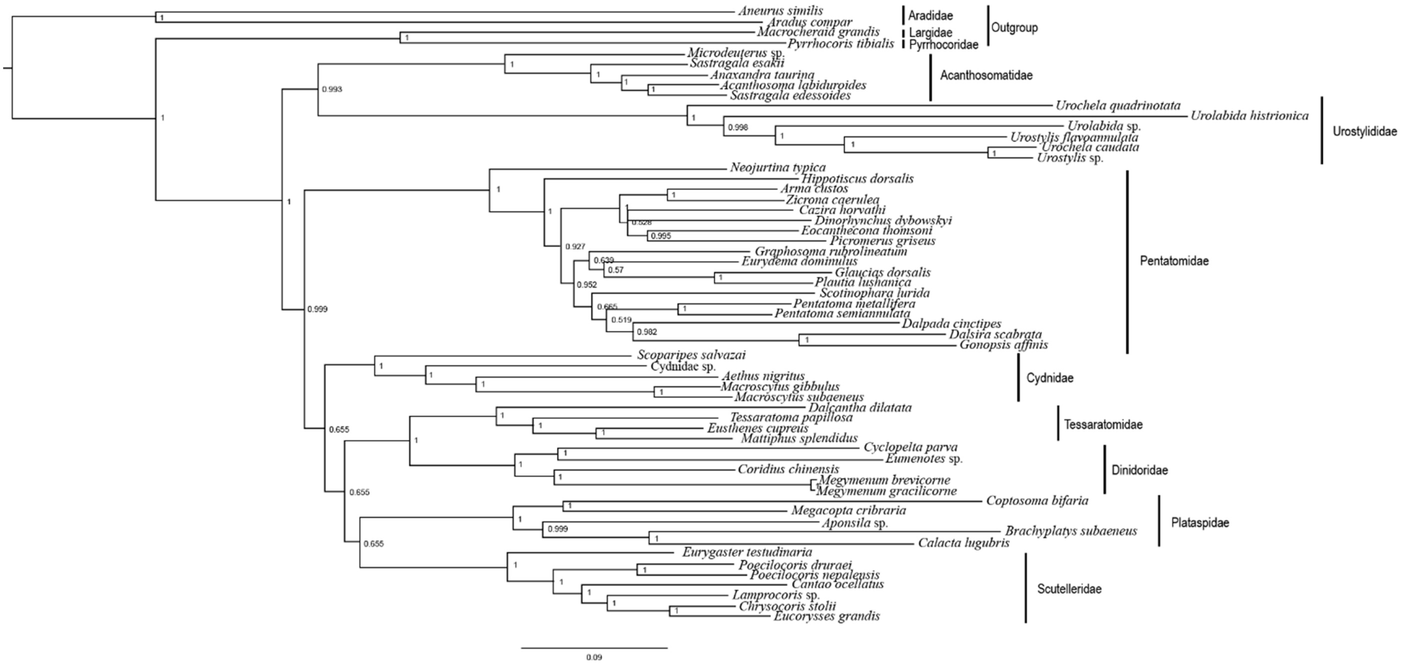

Figure S7. Phylogenetic tree of Pentatomoidea inferred via MrBayes based on the PCG12RNA dataset. The nodal support indicates the Bayesian posterior probabilities.

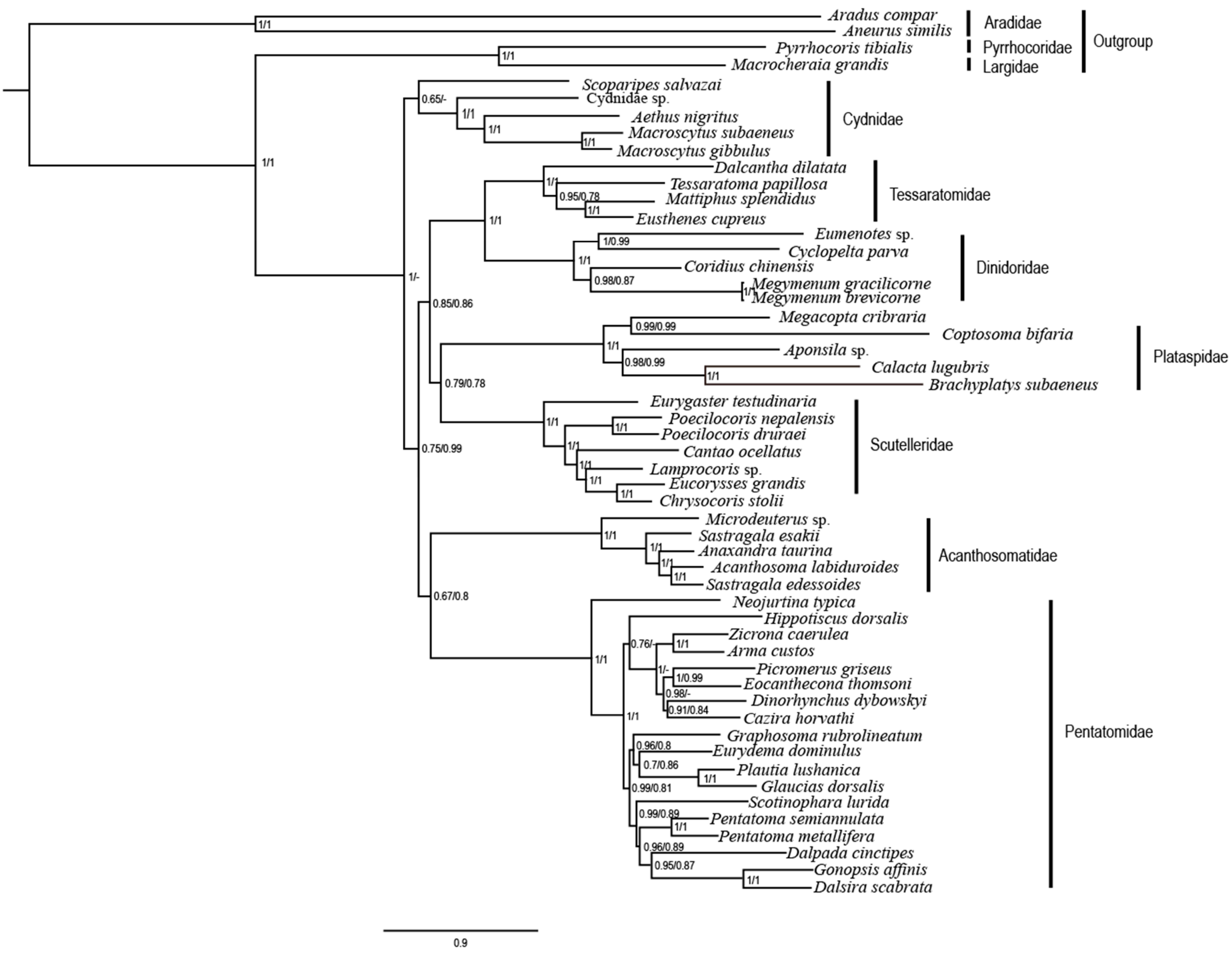

Figure S8. Phylogenetic tree of Pentatomoidea (excluding Urostylididae) inferred via PhyloBayes based on the PCGRNA and PCG12RNA datasets. The nodal support indicates the Bayesian posterior probabilities of PCGRNA/PCG12RNA. “-” indicates node support values below 0.50.

Table S1. Collection information of taxa used in the present study.

Name	Locality	Storage number	Time
Dalcantha dilatata	Mengla, Yunnan, China (99.559504 E, 23.022333 N)	CAU-HE-0001270	04, August 2013
Sastragala esakii	Guanshan National Geopark, Huixian, Xinxiang, Henan, China (113.527878 E, 35.565422 N)	CAU-HE-0000818	03, August 2018
Microdeuterus sp.	Jungle Girl Camp, Mt. Trus Madi, Kota Kinabalu, Malaysia Sabah (116.4515 E, 5.4433 N)	CAU-HE-0000379	22, July 2018
Aethus nigritus	West Campus, China Agricultural University, Haidian, Beijing, China (116.35832 E, 40.00433 N)	CAU-HE-0001580	18, April 2019
Macroscytus subaeneus	West Campus, China Agricultural University, Haidian, Beijing, China (116.35832 E, 40.00433 N)	CAU-HE-0001582	18, April 2019
Eumenotes sp.	Chinese Academy of Tropical Agricultural Sciences, Longhua, Haikou, Hainan, China (110.324425 E, 19.985543 N)	CAU-HE-0002745	21, May 2019
Poecilocoris druraei	Yuquanba Village, Qingmuchuan Ningqiang, Hanzhong, Shaanxi, China (105.67 E, 32.86 N)	CAU-HE-0001299	17, September 2018
Chrysocoris stolii	Tongfu to Shuikou Village, Nandao, Lidao, Hong Kong, China (113.920729 E, 22.223288 N)	CAU-HE-0002469	13, May 2019
Brachyplatys subaeneus	Tongfu to Shuikou Village, Nandao, Islands District, Hong Kong, China (113.920729 E, 22.223288 N)	CAU-HE-0002471	13, May 2019
Calacta lugubris	Tong Tsai Country Trail, Islands District, Hong Kong, China (113.932966 E, 22.256929 N)	CAU-HE-0002313	05, May 2019
Urochela caudata	Small dock, Muyu, Qingchuan, Sichuan, China (105.43 E, 32.66 N)	CAU-HE-0000096	28, April 2018
Urolabida histrionica	Lianzhu Town, Mojiang, Pu 'er, Yunnan, China (101.625269 E, 23.409103 N)	CAU-HE-0003243	29, July 2020
Glaucias dorsalis	Hengshitang, Shimentai Nature Reserve, Guangdong, China (113.378265 E, 24.347375 N)	CAU-HE-0001272	22, June 2017
Dalpada cinctipes	Sanjiaoshan National Forest Park, Xishui, Hubei, China (115.569929 E, 30.487660 N)	CAU-HE-0001230	10,July 2007
Eurydema dominulus	Liangjia Village, Qifang, Zaoyang, Xiangyang, Hubei, China (112.575746 E, 32.170055 N)	CAU-HE-0000779	14, June 2018
Neojurtina typica	Hengshitang, Shimentai Nature Reserve, Guangdong, China (113.378265 E, 24.347375 N)	CAU-HE-0000566	22, June 2017
Plautia lushanica	Guanshan National Geopark, Huixian, Xinxiang, Henan, China (113.527878 E, 35.565422 N)	CAU-HE-0000830	03, August 2018
Hippotiscus dorsalis	Fenghuang Lake, University of Chuzhou, Chuzhou, Anhui, China (118.313936 E,32.276030 N)	CAU-HE-0001790	27, June 2019
Pentatoma metallifera	The Water Great Wall, Huairou, Beijing, China (116.300691 N, 40.413169)	CAU-HE-0002084	19, August 2016
Zicrona caerulea	Xiaocaoba, Yiliang, Zhaotong, Yunnan, China (104.349712 E, 27.780037 N)	CAU-HE-0003242	09, July 2020

Table S2. Partition strategies used in phylogenetic analyses under site-homogeneous models.

Software	Dataset and Partition type	Subset Partitions
MrBayes	PCG12RNA<br>gene partition	**P1:** (ND2, ATP8, ND6, ATP6, ND3)
		**P2:** (COI)
		**P3:** (CYTB, COIII, COII)
		**P4:** (ND4, ND5, ND4L, ND1)
		**P5:** (srRNA, lrRNA)
MrBayes	PCGRNA<br>gene partition	**P1:** (ND6, ND2, ATP8)
		**P2:** (COI)
		**P3:** (CYTB, COIII, COII)
		**P4:** (ATP6, ND3)
		**P5:** (ND4, ND5, ND4L)
		**P6:** (ND1)
		**P7:** (lrRNA, srRNA)
IQ-TREE	PCG12RNA<br>gene partition	**P1:** (ND2, ATP8, ND6, ATP6, ND3)
		**P2:** (COI)
		**P3:** (CYTB, COIII, COII)
		**P4:** (ND4, ND5, ND4L, ND1)
		**P5:** (srRNA, lrRNA)
IQ-TREE	PCGRNA<br>gene partition	**P1:** (ND6, ND2, ATP8)
		**P2:** (COI)
		**P3:** (CYTB, COIII, COII)
		**P4:** (ATP6, ND3)
		**P5:** (ND4, ND5, ND4L)
		**P6:** (ND1)
		**P7:** (lrRNA, srRNA)
Mrbayes	PCG12RNA (excluding Urostylididae)<br>gene partition	**P1:** (ND2, ATP8, ND6, ATP6, ND3)
		**P2:** (COI)
		**P3:** (CYTB, COIII, COII)
		**P4:** (ND4, ND5, ND4L, ND1)
		**P5:** (srRNA, lrRNA)
Mrbayes	PCGRNA (excluding Urostylididae)<br>gene partition	**P1:** (ND6, ND2, ATP8)
		**P2:** (COI)
		**P3:** (CYTB, COIII, COII)
		**P4:** (ATP6, ND3)
		**P5:** (ND4, ND5, ND4L)
		**P6:** (ND1)
		**P7:** (lrRNA, srRNA)
IQ-TREE	PCG12RNA (excluding Urostylididae)<br>gene partition	**P1:** (ND2, ATP8, ND6, ATP6, ND3)
		**P2:** (COI)
		**P3:** (CYTB, COIII, COII)
		**P4:** (ND4, ND5, ND4L, ND1)
		**P5:** (srRNA, lrRNA)
IQ-TREE	PCGRNA (excluding Urostylididae)<br>gene partition	**P1:** (ND6, ND2, ATP8)
		**P2:** (COI)
		**P3:** (CYTB, COIII, COII)
		**P4:** (ATP6, ND3)
		**P5:** (ND4, ND5, ND4L)
		**P6:** (ND1)
		**P7:** (lrRNA, srRNA)
